# Mitral Valve Surgery with and Without Mitral Annular Disjunction: A Meta-Analysis

**DOI:** 10.3390/jcdd12110436

**Published:** 2025-11-04

**Authors:** Massimo Baudo, Francesco Cabrucci, Francesca Maria Di Muro, Dimitrios E. Magouliotis, Beatrice Bacchi, Arian Arjomandi Rad, Andrew Xanthopoulos, Tulio Caldonazo, Michele D’Alonzo

**Affiliations:** 1 Department of Cardiac Surgery Research, Lankenau Institute for Medical Research, Main Line Health, Wynnewood, PA 19096, USA; francesco.cabrucci.6@gmail.com (F.C.); magouliotisd@mlhs.org (D.E.M.); 2 Division of Cardiovascular Surgery, Peter Munk Cardiac Centre, Toronto General Hospital, University Health Network, Toronto, ON M5G 2C4, Canada; 3 Department of Medicine, Surgery and Dentistry, University of Salerno, 84084 Salerno, Italy; fdimuro94@gmail.com; 4 Division of Cardiac Surgery, St. Michael’s Hospital, University of Toronto, Toronto, ON M5S 1A1, Canada; beatricebacc@gmail.com; 5 Department of Cardiothoracic Surgery, John Radcliffe Hospital, Oxford University Hospitals NHS Foundation Trust, Oxford OX3 9DU, UK; arian.arjomandirad@gmail.com; 6 Department of Cardiology, University Hospital of Larissa, 41110 Larissa, Greece; andrewvxanth@gmail.com; 7 Department of Cardiothoracic Surgery, Jena University Hospital, 07747 Jena, Germany; tulio.caldonazo@med.uni-jena.de; 8 Cardiac Surgery Unit, Poliambulanza Foundation Hospital, Via Bissolati 57, 25124 Brescia, Italy; michele.dalonzo@poliambulanza.it

**Keywords:** cardiac surgery, mitral valve surgery, mitral annular disjunction, ventricular arrhythmia, meta-analysis

## Abstract

Background/Objectives: Despite growing awareness of mitral annular disjunction’s (MAD) clinical significance, robust data regarding the outcomes of surgical interventions on MAD remain sparse. This meta-analysis aims to systematically evaluate the current evidence on the efficacy and safety of mitral valve (MV) surgery in patients with MAD. Methods: A systematic review was conducted from inception until May 2025 for studies comparing patients undergoing MV surgery with and without MAD and was registered in PROSPERO: CRD42025649821. Results: Patients with MAD were generally younger (59.3 ± 5.0 vs. 63.4 ± 2.2 years, standardized mean difference: −0.3073), had fewer comorbidities but more complex valve lesions (41.0% vs. 13.7%, risk difference: 0.2627) compared to those without MAD. MV replacement was performed less frequently in the MAD group than in the No-MAD group (risk ratio, RR: 0.690 [95% confidence interval, CI: 0.508; 0.937], *p* = 0.017), probably related to the significant difference in age between the two groups. The MAD cohort demonstrated a higher incidence of ventricular arrhythmia both after surgery (RR: 7.255 [95%CI: 1.231; 42.763], *p* = 0.029) and during follow-up (incidence rate ratio, IRR: 2.750 [95%CI: 1.372; 5.512], *p* = 0.004). Although the MAD group experienced more arrhythmic events over time, this did not translate into a significant difference in overall mortality compared to patients without MAD (IRR: 0.573 [95%CI: 0.072; 4.555], *p* = 0.599). Conclusions: This meta-analysis revealed significant baseline differences between the populations. Our findings suggest that patients with MAD remained at significantly higher risk for both postoperative and long-term arrhythmias. These results highlight the need for close arrhythmic surveillance in this population.

## 1. Introduction

Mitral annular disjunction (MAD) is an increasingly recognized structural abnormality of the mitral valve (MV), characterized by an atypical spatial separation between the mitral annulus, particularly its posterior segment, and the basal portion of the left ventricular (LV) myocardium [1]. The circumferential spread of MAD is constrained on the anterior side by the mitro-aortic continuity [2]. Therefore, MAD is typically confined to the region of the posterior leaflet, where it may extend to varying degrees beneath its scallops, most commonly beneath the central one. Rather than forming a continuous fibromuscular unit, as typically expected, the annulus in MAD is displaced toward the left atrium, resulting in a hypermobile annular segment during the cardiac cycle. This decoupling disrupts the synchronized motion of the MV apparatus, giving rise to the phenomenon known as “annular curling” and contributing to mechanical instability within the mitral valve–left ventricle interface [3].

Initially described in 1876 by Henle [4], it was first given the term “disjunction” in autopsy studies during the 1980s [5]. Thereafter, MAD remained underappreciated for several decades due to limited imaging modalities and unclear clinical implications. However, the advent and refinement of advanced echocardiographic techniques and cardiac magnetic resonance have greatly improved the detection and characterization of MAD, especially in the context of mitral valve prolapse (MVP) [6]. Recent data indicate that MAD is present in up to 40% of MVP cases, particularly in patients with myxomatous degeneration, while its prevalence in the general population ranges from 7% to 9% [7].

From a pathophysiological standpoint, MAD is no longer regarded as a benign incidental finding. Instead, it is now recognized as a potential substrate for serious ventricular arrhythmias (VA), including, in some cases, sudden cardiac death (SCD) [8]. These arrhythmogenic events are thought to arise from increased mechanical stress and abnormal strain distribution across the mitral leaflets, chordae tendineae, and papillary muscles, often accompanied by localized fibrosis [9]. The concept of arrhythmic MVP has emerged to describe a subset of patients with MVP and underlying MAD who are particularly vulnerable to malignant arrhythmias [10]. MAD has been implicated in VA both with and without MVP [11]. Meta-analytic data show that MVP patients with MAD have nearly twice the risk of arrhythmias [12].

The natural history of MAD remains incompletely understood, particularly regarding its progression and impact following surgical intervention. Medically managed MAD carries worse arrhythmia-free survival than controls [13]. It has been hypothesized that failure to recognize and address MAD intraoperatively may compromise the long-term durability of MV repair and leave residual substrates for arrhythmogenesis [14]. While MV surgery can address mitral regurgitation (MR) and potentially reduce annular mobility, its ability to fully correct the structural disjunction remains uncertain. Some studies suggest that even after surgical correction of MR, patients with MAD may continue to exhibit an elevated arrhythmic risk, possibly due to irreversible myocardial remodeling or an underlying cardiomyopathic process [15,16,17]. Early reports also suggest that intraoperative papillary muscle cryoablation, performed during mitral surgery, may reduce ectopy [18,19,20,21].

Moreover, the anatomical variability of MAD, now sub-classified into “true” (persistent in both systole and diastole) and “pseudo” (systolic-only) forms, adds to the complexity of clinical decision-making and surgical planning [22].

Despite growing awareness of MAD’s clinical significance, robust data regarding the outcomes of surgical interventions specifically targeting MAD remain sparse. This meta-analysis aims to systematically evaluate the current evidence on the efficacy and safety of MV surgery in patients with MAD, with a particular focus on arrhythmic outcomes, structural correction, and long-term survival. By synthesizing available literature, this meta-analysis seeks to clarify the role of surgical management in this complex and evolving condition and to identify gaps that warrant further investigation.

## 2. Materials and Methods

This systematic review was registered in the PROSPERO database under the identifier CRD42025649821. Since all data used were sourced from prior published literature and did not involve direct human subject interaction or access to personally identifiable information, neither ethical committee approval nor informed consent was required. Data analyzed during the review are available from the corresponding author upon reasonable request.

### 2.1. Literature Search and Study Selection Criteria

**Figure 1:**
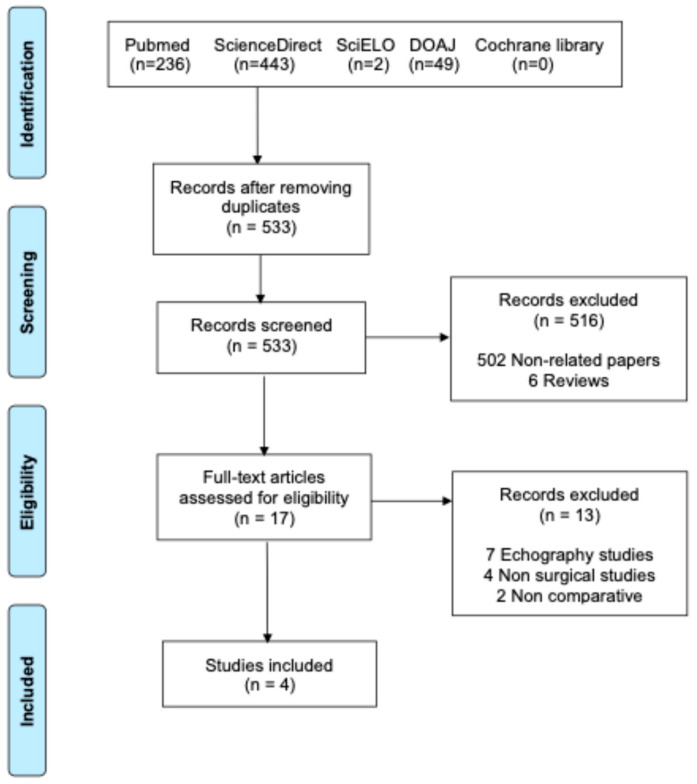
PRISMA flowchart of the included studies.

A thorough literature search strategy was performed in accordance with the PRISMA (Preferred Reporting Items for Systematic Reviews and Meta-Analyses) guidelines [23]. The PRISMA flow chart of study selection is illustrated in Figure 1. The literature search queried several databases, including PubMed, ScienceDirect, DOAJ, SciELO, and the Cochrane Library, from inception through May 2025 for comparative studies assessing outcomes in patients undergoing MV surgery with and without MAD.

The search was framed using the PICOS model: (1) Population: Adults (aged 18 and over) undergoing surgical intervention for MR; (2) Intervention: Surgical correction of MR with concurrent MAD; (3) Comparator: Surgical correction of MR without MAD; (4) Outcomes: Clinical outcomes with particular attention to ventricular arrhythmias; (5) Study Design: Randomized controlled trials and both prospective and retrospective observational studies.

Additionally, backward citation tracking (i.e., backward snowballing) was performed to capture any potentially relevant studies not identified through database queries. The complete search algorithm is detailed in Supplemental Table S1.

Three independent reviewers (M.B., F.C., and D.E.M.) performed title, abstract, and full-text screening. Disagreements were resolved collaboratively through discussion until consensus was achieved.

Only studies published in English were considered. Exclusion criteria included abstracts without full articles, editorials, conference proceedings, commentaries, case reports, case series, and review articles. In situations where multiple publications appeared to originate from the same research group or cohort, we reviewed the data collection periods to identify any overlapping populations. In such cases, the study featuring the largest patient cohort was selected for inclusion.

The methodological quality of the included studies was assessed using the ROBINS-I tool [24] for non-randomized studies and the RoB 2 tool [25] for RCTs.

### 2.2. Data Extraction and Quality Assessment

Data were systematically extracted into Microsoft Excel (Office 365, Microsoft Corp., Redmond, WA, USA). Binary outcomes were tabulated as counts and percentages, whereas continuous variables were summarized as reported in the source articles (mean or median, standard deviation, range, or interquartile range). When necessary, means were estimated from medians and other descriptive statistics using the method developed by Luo and colleagues [26]. Extracted variables included key study-level details such as setting, design, duration, and sample size. Given the diversity in reporting formats across studies, harmonization of variables and standardization procedures were applied to allow for uniform comparisons. The author’s definition of VA was used for each included study, as well as for MAD. Different MAD thresholds were utilized, from any visible separation (>0 mm) [27], ≥3 mm [28], >5 mm [14,28], or >8.5 mm [29].

### 2.3. Statistical Analysis

To address heterogeneity in reported baseline continuous variables, standardization to mean and standard deviation was performed using the method for unknown non-normal distributions (MLN) proposed by Cai et al. [30]. Summary estimates were weighted by sample size, and comparisons between groups were made using standardized mean differences (SMD) for continuous outcomes and risk differences (RD) for categorical outcomes.

Comparative statistics included the calculation of risk ratios (RR) or SMD with corresponding 95% confidence intervals (CI) for dichotomous and continuous outcomes, respectively. For single-arm outcomes, pooled event rates (PER) and pooled mean estimates (PEM) were calculated. To address late outcomes, Poisson regression modeling was employed, which adjusts for variations in follow-up durations across studies by assuming a constant event rate. The cumulative person-time was derived based on the number of events and the average follow-up period [31]. A logarithmic transformation was applied to estimate the overall incidence rate ratio (IRR), and a random effects model was incorporated. A random-effects model using the inverse variance weighting with the DerSimonian–Laird method was employed to accommodate heterogeneity across studies. The No-MAD group was set as the reference for all comparisons. Studies reporting zero events were adjusted using a continuity correction.

Statistical heterogeneity was evaluated using Cochran’s Q test and the I^2^ statistic. Due to the limited number of studies (<10), Egger’s test for publication bias was not performed.

All analyses were conducted using R software (version 4.4.3) in the RStudio environment. A *p*-value less than 0.05 was considered indicative of statistical significance.

## 3. Results

Figure 1 illustrates the PRISMA flowchart in conducting the systematic review. The initial search yielded 730 records. After eliminating duplicates, 533 unique studies remained and were subjected to screening. Of these, 17 articles were reviewed in full to determine their suitability. Ultimately, four studies [14,27,28,29] met the predefined inclusion criteria, encompassing a combined total of 995 participants: 238 in the MAD cohort and 757 in the No-MAD cohort. These studies, all published between 2022 and 2025, are summarized in Table 1. All studies were retrospective, one of which was propensity score matched [29]. The critical appraisal for the included studies is presented in Supplemental Table S2.

Patients in the MAD group were younger than those in the No-MAD group (mean age 59.3 ± 5.0 vs. 63.4 ± 2.2 years; SMD = −0.3073). They also had a lower prevalence of hypertension (25.2% vs. 33.2%; RD = −0.1051) and were less likely to exhibit severe symptoms (31.3% vs. 49.2%; RD = −0.1496). Conversely, bileaflet MV prolapse was more common in the MAD group (41.0% vs. 13.7%; RD = 0.2627). A detailed summary of baseline characteristics is provided in Table 2.

### Meta-Analysis

Intraoperatively, patients in the MAD group underwent mitral valve replacement (MVR) less frequently than those in the No-MAD group (RR: 0.690; 95% CI: 0.508–0.937; *p* = 0.017), Figure S1. No other significant intraoperative differences were observed between the groups.

Postoperatively, the MAD group showed a higher incidence of ventricular arrhythmias compared to the No-MAD group (RR: 7.255; 95% CI: 1.231–42.763; *p* = 0.029), Figure S2. Residual MAD was present in 1.9% of patients (95% CI: 0.7–5.3%), Figure S3. Aside from this, no additional significant postoperative differences were reported.

During a mean follow-up of 3.5 years in both groups (Figures S4 and S5), the MAD group continued to exhibit a higher rate of ventricular arrhythmia events (IRR: 2.750; 95% CI: 1.372–5.512; *p* = 0.004), Figure S6. The remaining forest plots are displayed in Figures S7–S19 (Table 3).

## 4. Discussion

This meta-analysis represents a comprehensive synthesis of current evidence evaluating surgical outcomes in patients with MAD, with particular attention to arrhythmic risk. The key findings of this meta-analysis can be summarized as follows: (1) Patients with MAD who underwent MV surgery were generally younger, had fewer comorbidities but more complex valve lesions compared to those without MAD. (2) MVR was performed less frequently in the MAD group than in the No-MAD group, probably related to the significant difference in age between the two groups. (3) The MAD cohort demonstrated a higher incidence of VA both after surgery and during follow-up. (4) Although the MAD group experienced more arrhythmic events over time, this did not translate into a significant difference in overall mortality compared to patients without MAD.

Patients with MVP and MAD exhibit distinct patterns of LV remodeling, characterized by disproportionately enlarged LV dimensions not explained by MR severity or age [13]. This may result from mechanical de-anchoring of the annulus, inefficient contraction, localized myocardial fibrosis or atrophy, and possibly underlying genetic or cardiomyopathic substrates, although a definitive genotype-phenotype association with MAD remains unproven [1]. Structural abnormalities of the mitral annulus often precede the onset of significant MR in MVP, suggesting that annular pathology may be a primary driver of disease development [32]. MAD has emerged as an important structural feature associated with an increased risk of VA, particularly in patients with MVP [33,34]. While the overall incidence of SCD in MVP remains low [35,36], MAD appears to contribute independently to arrhythmogenic risk through the previously described mechanisms of mechanical stretch, myocardial fibrosis, and structural remodeling [37]. However, current major guidelines do not recommend ICD implantation solely for the presence of MAD. Decisions follow the usual secondary-prevention indications, and individualized risk assessment for primary prevention remains the rule. The EHRA 2022 consensus paper on arrhythmic MVP/MAD emphasizes phenotype-guided risk stratification, which includes MAD among others [2]. Imaging studies have further linked MAD to inferolateral myocardial fibrosis, a known arrhythmic substrate [38,39]. Moreover, a greater degree of annular displacement (MAD length > 8.5 mm) has been correlated with higher arrhythmic risk and the presence of myocardial fibrosis [8]. The presence of MAD in MVP patients is now widely considered a marker of arrhythmic vulnerability, independent of conventional risk factors such as MR severity or LV dysfunction, although MAD does not consistently predict mortality [40]. This last point was confirmed by the present analysis, in which overall mortality rates were comparable between MAD and No-MAD patients. Nevertheless, after 10 years from MAD diagnosis, the subsequent onset of arrhythmias is independently associated with an increased risk of death [41]. Such long-term follow-up data were not available in the included studies and could not be evaluated in the current study. This delayed arrhythmic manifestation may reflect progressive myocardial fibrosis in patients with the MAD-MVP phenotype, which evolves over time and contributes to later electrical instability. Indeed, the association of MAD with the gradual emergence of ventricular arrhythmias is well established [13]. A recent meta-analysis confirmed that the presence of MAD was found to markedly increase the likelihood of developing complex VA, with affected patients showing nearly a fourfold higher risk compared with those without MAD [42]. Moreover, patients with MAD faced an elevated risk for the combined endpoint of complex VA and SCD. Evidence indicates that patients with MAD, even in the absence of baseline arrhythmias, face a higher risk of developing significant ventricular arrhythmias over time [41]. However, the natural history of MAD remains poorly understood, and longitudinal data on the anatomical progression of MAD are scarce. Therefore, limitations in study size and methodological variability across the literature leave room for uncertainty regarding the exact role of MAD in predicting SCD.

MAD is primarily diagnosed through cardiovascular imaging modalities such as transthoracic (TTE) and transesophageal echocardiography (TEE), cardiac computed tomography (CT), and cardiovascular magnetic resonance (CMR) [43]. TTE remains the first-line, non-invasive modality for identifying MAD and assessing associated hemodynamic effects, while TEE, particularly with 3D imaging, offers higher spatial resolution and detailed anatomical visualization [44]. Cardiac CT provides excellent spatial detail of the mitral annulus but lacks real-time functional assessment, serving mainly as an adjunct tool [45]. CMR, regarded as the gold standard, enables precise measurement of MAD, detection of myocardial fibrosis via late gadolinium enhancement, and comprehensive evaluation of LV morphology and function [45]. Comparative studies show that CMR detects MAD more frequently than echocardiography, with stronger agreement between TEE and CMR than between TTE and CMR [46]. Overall, despite advances in imaging, heterogeneity in MAD definitions and measurement techniques continues to limit diagnostic consistency and risk stratification, underscoring the need for standardized imaging protocols [43].

Clinical management of MAD remains challenging due to limited evidence. Medical therapy, primarily beta-blockers and antiarrhythmic agents, may help reduce ventricular arrhythmia burden in selected patients, although no randomized trials have been conducted in the MAD population [47]. Catheter ablation targeting arrhythmic foci has shown promising outcomes in symptom reduction and improved LV function, though recurrence rates remain high (26–32%) due to procedural challenges and ongoing myocardial remodeling [48,49,50]. For VA originating from papillary muscles, catheter ablation achieves high acute success (85–93%), but long-term single-procedure freedom from recurrence is 67–79%, often requiring repeat procedures [51,52,53]. Surgical papillary muscle cryoablation performed concomitantly with mitral repair has emerged as an option in selected patients (e.g., clear papillary muscle focus, failed catheter ablation, or when surgery is already indicated), with favorable case-series durability under direct visualization [18,19,20,21]. Nevertheless, robust comparative trials are lacking. Therefore, surgery has been proposed as a valid solution.

MV surgery is recommended for patients with MVP and severe MR [54,55]. It has been hypothesized that in cases with MAD, surgical correction using a prosthetic ring to reattach the annulus to the LV (either through annuloplasty or using a prosthetic valve) may reduce mechanical stress on the papillary muscles and lower the risk of ventricular arrhythmias [13,41,56]. This would benefit especially younger patients, potentially due to less advanced myocardial fibrosis at the time of intervention [57]. In older patients or those with established fibrosis, the anti-arrhythmic benefit may be limited. Evidence from the present meta-analysis directly comparing patients with and without MAD has shown that even after successful surgical correction of MAD and despite being younger in age, patients with preoperative MAD experienced a significantly elevated postoperative and long-term risk of developing VA following MV surgery. Although MV surgery, repair and replacement can anatomically correct MAD (<2% residual MAD was reported in this analysis), the pre-existing mechanical strain on the chordae and papillary muscles may have already led to myocardial fibrosis, creating an arrhythmogenic substrate that persists postoperatively and after discharge [58]. Lodin et al. have argued that residual arrhythmic risk may also result from suboptimal surgical technique, such as inadequate placement or tensioning of artificial chordae, mismatched annuloplasty rings, or continued progression of Barlow’s disease and adverse left ventricular remodeling [28]. These findings highlight that successful valve surgery does not necessarily eliminate the risk of VA in this population, underlining the importance of ongoing surveillance and individualized management strategies. Currently, the only arrhythmia concomitant to MV disease for which MV surgery is indicated is atrial fibrillation (Class IIa) [55].

Regarding the “repairability” of the MV in patients with MAD, some have proposed that MAD could compromise the durability of the repair by contributing to abnormal systolic annular motion, including paradoxical expansion and flattening [59,60]. This was confirmed by Biondi et al., who observed two distinct morphological patterns of MAD in patients undergoing MV surgery: a bimodal configuration and a uniformly distributed form [61]. The bimodal variant showed reduced disjunction distance along the central segment of the annulus, whereas the uniform type exhibited a more consistent separation throughout. These findings suggest that MAD represents a spectrum of structural variations rather than a simple binary condition, consisting of two predominant phenotypes. Despite this anatomical heterogeneity, the presence of MAD did not increase surgical complexity. Patients with MAD are generally associated with more complex valves, with extensive leaflet scallop involvement, more frequent bileaflet prolapse, larger annular dimensions and higher degrees of regurgitant fraction [6,41]. Our meta-analysis confirmed that patients with MAD had a higher prevalence of bileaflet lesions in the operated valves, but they presented lower rates of MVR compared to those without MAD. This difference likely reflects the significant age disparity between the two groups: younger patients (MAD) are typically offered valve repair to ensure greater long-term durability, whereas older patients (No-MAD) are more often treated with valve replacement. All included studies consistently reported excellent mitral valve repair outcomes in the MAD group, although not quantifiable in this analysis, that were comparable to those observed in patients without MAD. Therefore, mitral valve repair in patients with MAD is generally feasible, including in bileaflet disease, and MAD does not compromise reparability. What is distinctive in MAD is the need to re-establish annular continuity with the LV myocardium: when annuloplasty sutures are placed at the ventricular myocardium level, the ring effectively “reattaches” the annulus and closes the MAD gap, while standard leaflet techniques address any degenerative pathology. This annular repositioning cannot be achieved with transcatheter edge-to-edge repair [62,63]. Essayagh et al. showed that the disjunction between the annulus and adjacent myocardium produces a misleading appearance of strong left ventricular contraction, which does not translate into true functional improvement following surgical repair [59]. Although MAD does not preclude successful valve reconstruction, it necessitates meticulous anchoring of the annuloplasty ring to the ventricular myocardium to prevent residual disjunction after surgery.

### Limitations

This meta-analysis has several important limitations to acknowledge. First, all included studies were retrospective in nature, with only one employing propensity score matching, and most lacked adjustment for confounders. This introduces a considerable risk of bias due to baseline imbalances between groups, particularly as patients with MAD were generally younger, a factor that may also influence the choice between mitral valve repair and replacement, thereby contributing to selection bias. In addition, patients with MAD may have undergone more intensive (rhythm) surveillance compared with those without MAD, potentially leading to over-detection of VA (surveillance bias). This could have influenced the observed association between MAD and VA. As such, these findings should be viewed as hypothesis-generating rather than definitive evidence to inform clinical practice. Furthermore, given the limited research available on this condition, only a small number of studies directly comparing patients with and without MAD met the inclusion criteria, which constrains the generalizability of our conclusions. Moreover, data on ECG or MRI findings, as well as other key MAD-related events, were not reported by more than one study, if at all. Consequently, these variables could not be included in the analysis. Finally, interpretation of the findings is further hindered by the absence of a uniform definition for significant end-systolic MAD length, as previous studies have applied thresholds ranging from any visible separation to as high as 8.5 mm.

## 5. Conclusions

This meta-analysis comparing outcomes of mitral valve surgery in patients with and without MAD revealed significant baseline differences between the two groups. Although prior studies have proposed that surgery may alleviate mechanical stress on the papillary muscles and reduce the risk of ventricular arrhythmias, our findings suggest otherwise: patients with MAD remained at significantly higher risk for both postoperative and long-term arrhythmias. These results highlight the need for close arrhythmic surveillance in this population. Larger, prospective studies are needed to validate these findings.

## Figures and Tables

**Table 1:** Included studies.

Paper	Study Period	Study	Institution	Country
Bennett 2022 [27]	2013–2020	OBS	Heart and Lung Centre, University Hospitals of North Midlands	UK
Gray 2023 [14]	January 2016–April 2020	OBS	Prince of Wales Hospital, Sydney, NSW	Australia
Muneretto 2025 [29]	January 2021–December 2023	PSM	ASST Spedali Civili di Brescia, Brescia	Italy
Lodin 2025 [28]	2010–2022	OBS	Karolinska University Hospital, Stockholm	Sweden

OBS = retrospective observational study; PSM = propensity score matched study.

**Table 2:** Baseline patient characteristics of the included studies.

Characteristic	MAD	No-MAD	SMD/RD
Age, years	59.3 ± 5.0	63.4 ± 2.2	SMD: −0.3073
Male sex	66.4% (158/238)	77.7% (588/757)	RD: −0.0507
BMI, kg/m^2^	25.0 ± 2.6	25.2 ± 0.8	SMD: −0.0588
Diabetes	2.5% (6/238)	3.8% (29/757)	RD: −0.0144
Dyslipidemia	15.5% (37/238)	16.2% (123/757)	RD: −0.0690
History of smoke	32.4% (77/238)	34.6% (262/757)	RD: −0.0693
Chronic lung disease	3.8% (6/156)	5.6% (35/628)	RD: −0.0211
Hypertension	25.2% (60/238)	33.2% (251/757)	RD: −0.1051
Heart failure	13.0% (12/92)	15.2% (31/204)	RD: −0.0127
NYHA III–IV	31.3% (40/128)	49.2% (287/582)	RD: −0.1496
CVA	1.7% (4/238)	4.2% (32/757)	RD: −0.0242
CAD	12.0% (11/92)	15.7% (32/204)	RD: −0.0423
Previous PCI	4.3% (4/92)	7.8% (16/204)	RD: −0.0353
PAD	2.2% (4/178)	1.9% (12/632)	RD: −0.0111
Creatinine	0.92 ± 0.22	1.00 ± 0.31	RD: −0.2331
Atrial fibrillation	23.5% (56/238)	32.0% (242/757)	RD: −0.0432
History of VA	6.1% (5/82)	3.9% (5/129)	RD: −0.0099
Ejection fraction	59.8 ± 7.2	59.2 ± 7.3	SMD: 0.0151
MAD length, mm	9.0 ± 3.6	-	-
MVP			
Anterior	4.3% (8/188)	13.9% (98/707)	RD: −0.1201
Posterior	51.6% (97/188)	67.5% (477/707)	RD: −0.1181
Bileaflet	41.0% (77/188)	13.7% (97/707)	RD: 0.2627
EuroSCORE II	2.17 ± 0.49	2.82 ± 1.22	SMD: −0.0714
Reintervention	0% (0/142)	2.0% (5/254)	RD: −0.0148

The denominator is based on the data availability among the included studies. BMI = body mass index; CAD = coronary artery disease; CVA = cerebrovascular accident; MAD = mitral annular disjunction; MVP = mitral valve prolapse; NYHA = New York Heart Association; PAD = peripheral artery disease; PCI = percutaneous coronary intervention; RD = risk difference; SMD = standardized mean difference; VA = ventricular arrhythmia.

**Table 3:** Meta-analysis summary of the perioperative outcomes.

Outcome	No. Studies	No. Patients	Effect [95%CI], *p*-Value	MAD [95%CI]	No-MAD [95%CI]	Heterogeneity (I^2^, *p*-Value)
CPB time, min	2	699	^a^ 0.060 [−0.131; 0.251], 0.539	136.6 [121.1; 154.0]	132.9 [115.7; 152.6]	0.0%, *p* = 0.7327
CXC time, min	2	699	^a^ 0.046 [−0.145; 0.236], 0.640	101.3 [98.1; 104.7]	99.1 [95.9; 102.5]	0.0%, *p* = 0.7108
MVR	4	995	^b^ 0.690 [0.508; 0.937], **0.017**	14.5% [6.2; 30.3]	23.6% [11.3; 42.7]	0.0%. *p* = 0.7265
MV repair	4	995	^b^ 1.079 [0.983; 1.186], 0.111	85.5% [69.7; 93.8]	76.4% [57.3; 88.7]	64.9%, *p* = 0.0358
AF ablation	2	285	^b^ 0.959 [0.465; 1.978], 0.909	14.8% [9.3; 22.9]	14.9% [4.3; 40.8]	34.0%, *p* = 0.2183
LAAO	2	285	^b^ 1.037 [0.506; 2.128], 0.921	10.2% [5.7; 17.4]	9.8% [6.2; 15.2]	0.0%, *p* = 0.3691
CABG	3	396	^b^ 0.584 [0.330; 1.035], 0.065	9.1% [3.5; 21.3]	18.2% [12.8; 25.4]	0.0%, *p* = 0.5189
Aortic surgery	2	285	^b^ 0.577 [0.087; 3.823], 0.569	2.8% [0.9; 8.4]	5.2% [1.2; 19.2]	47.6%, *p* = 0.1672
Surgical revision	2	699	^b^ 0.528 [0.203; 1.370], 0.189	3.5% [1.4, 8.0]	6.0% [4.3; 8.3]	0.0%, *p* = 0.9377
POAF	2	211	^b^ 1.088 [0.681; 1.738], 0.725	34.2% [24.7; 45.0]	31.9% [19.0; 48.5]	21.5%, *p* = 0.2591
VA	2	211	^b^ 7.255 [1.231; 42.763], **0.029**	7.5% [3.4; 15.7]	1.2% [0.2; 5.6]	0.0%, *p* = 0.9761
Postop MAD	4	238	-	1.9% [0.7; 5.3]	-	0.0%, *p* = 0.6243
Mod/Sev MR	3	396	^b^ 0.851 [0.376; 1.929], 0.699	5.0% [1.4; 16.4]	3.6% [0.5; 20.9]	0.0%, *p* = 0.4368
Hospital mortality	3	810	^b^ 0.453 [0.040; 5.067], 0.520	0.9% [0.2; 4.4]	1.0% [0.5; 2.2]	0.0%, *p* = 0.8328
Follow-up years	4	995	-	3.5 [2.5; 5.0]	3.5 [2.7; 4.6]	-
FUP mortality	4	995	^c^ 0.573 [0.072; 4.555], 0.599	1.0%/yr [0.1; 9.2]	1.6%/yr [1.1; 2.5]	76.9%, *p* = 0.0046
FUP CVA	2	784	^c^ 0.933 [0.498; 1.745]. 0.827	1.6%/yr [0.9; 2.9]	1.8%/yr [1.3; 2.3]	0.0%, *p* = 0.4339
FUP VA	3	895	^c^ 2.750 [1.372; 5.512], **0.004**	2.0%/yr [0.4; 10.5]	1.1%/yr [0.5; 2.5]	0.0%, *p* = 0.1264

The estimate corresponds to (a) standardized mean difference, (b) risk ratio, (c) incidence rate ratio. Bold indicates *p* < 0.05. AF = atrial fibrillation; CABG = coronary artery bypass grafting; CPB = cardiopulmonary bypass; CVA = cerebrovascular accident; CXC = cross clamp; FUP = follow-up; LAAO = left atrial appendage occlusion; MAD = mitral annular disjunction; MR = mitral regurgitation; MV = mitral valve; MVR = mitral valve replacement; POAF = postoperative atrial fibrillation; VA = ventricular arrhythmia.

## Data Availability

Data compiled or analyzed throughout the study can be made available by the corresponding author upon reasonable request.

## Supplementary Materials

The following supporting information can be downloaded at: https://www.mdpi.com/article/10.3390/jcdd12110436/s1, PRISMA 2020 checklist. Figure S1: Pooled estimated risk ratio of mitral valve replacement, Figure S2: Pooled estimated risk ratio of postoperative ventricular arrhythmias, Figure S3: Pooled estimated rate of postoperative residual mitral annular disjunction, Figure S4: Pooled estimated mean of follow-up time for MAD patients, Figure S5: Pooled estimated mean of follow-up time for No-MAD patients, Figure S6: Pooled estimated incidence rate ratio of follow-up ventricular arrhythmias, Figure S7: Pooled estimated standardized mean difference for cardiopulmonary bypass time, Figure S8: Pooled estimated standardized mean difference for cross-clamp time, Figure S9: Pooled estimated risk ratio of mitral valve repair, Figure S10: Pooled estimated risk ratio of concomitant atrial fibrillation ablation, Figure S11: Pooled estimated risk ratio of concomitant left atrial appendage occlusion, Figure S12: Pooled estimated risk ratio of concomitant CABG, Figure S13: Pooled estimated risk ratio of concomitant aortic surgery, Figure S14: Pooled estimated risk ratio of surgical revision, Figure S15: Pooled estimated risk ratio of postoperative atrial fibrillation, Figure S16: Pooled estimated risk ratio of residual moderate/severe mitral regurgitation, Figure S17: Pooled estimated risk ratio of hospital mortality, Figure S18: Pooled estimated incidence rate ratio of follow-up mortality, Figure S19: Pooled estimated incidence rate ratio of cerebrovascular accidents; Table S1: Search strategy, Table S2: Risk of Bias in Non—Randomized Studies of Interventions (ROBINS-I) with traffic lights.

## Abbreviations

The following abbreviations are used in this manuscript:

CI	Confidence interval
IRR	Incidence rate ratio
LV	Left ventricle
MAD	Mitral annular disjunction
MV	Mitral valve
MVP	Mitral valve prolapse
MVR	Mitral valve replacement
SCD	Sudden cardiac death
SMD	Standardized mean difference
RD	Risk difference
RR	Risk ratio
