# Supplementary material for: Mitral Valve Surgery with and Without Mitral Annular Disjunction: A Meta-Analysis
Source: J Cardiovasc Dev Dis. 2025 Nov 4;12(11):436. doi: 10.3390/jcdd12110436 (PMC12653801; doi:10.3390/jcdd12110436)

Table S1 – Search strategy

#	Search
1	("mitral"[All Fields] OR "mitralic"[All Fields] OR "mitrals"[All Fields]) AND "annular"[All Fields] AND ("disjunct"[All Fields] OR "disjunction"[All Fields] OR "disjunctions"[All Fields] OR "disjunctive"[All Fields] OR "disjunctively"[All Fields] OR "disjuncts"[All Fields])
2	"surgery"[MeSH Subheading] OR "surgery"[All Fields] OR "surgical procedures, operative"[MeSH Terms] OR ("surgical"[All Fields] AND "procedures"[All Fields] AND "operative"[All Fields]) OR "operative surgical procedures"[All Fields] OR "general surgery"[MeSH Terms] OR ("general"[All Fields] AND "surgery"[All Fields]) OR "general surgery"[All Fields] OR "surgery s"[All Fields] OR "surgerys"[All Fields] OR "surgeries"[All Fields]
3	1 AND 2

Table S2 - Risk of Bias in Non-Randomized Studies of Interventions (ROBINS-I) with traffic lights

Study	D1	D2	D3	D4	D5	D6	D7	Overall
Bennett 2022	⊖	⊖	⊕	⊕	⊕	⊕	⊖	⊕
Gray 2023	⊖	⊖	⊕	⊕	⊕	⊕	⊖	⊕
Muneretto 2025	⊕	⊖	⊕	⊕	⊕	⊕	⊖	⊕
Lodin 2025	⊖	⊖	⊕	⊕	⊕	⊕	⊖	⊕

D1 = bias due to confounding; D2 = bias due to selection of participants; D3 = bias in classification of interventions; D4 = bias due to deviation from intended interventions; D5 = bias due to missing data; D6 = bias in measurements of outcomes; D7 = bias in selection of the reported results.

⊕ = low bias; ⊖ = moderate bias; ⊗ = serious bias.

Figure S1 – Pooled estimated risk ratio of mitral valve replacement

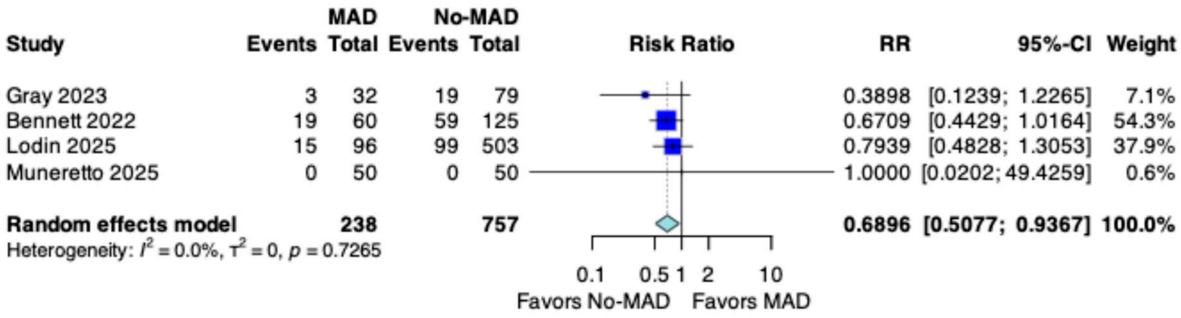

Figure S2 – Pooled estimated risk ratio of postoperative ventricular arrhythmias

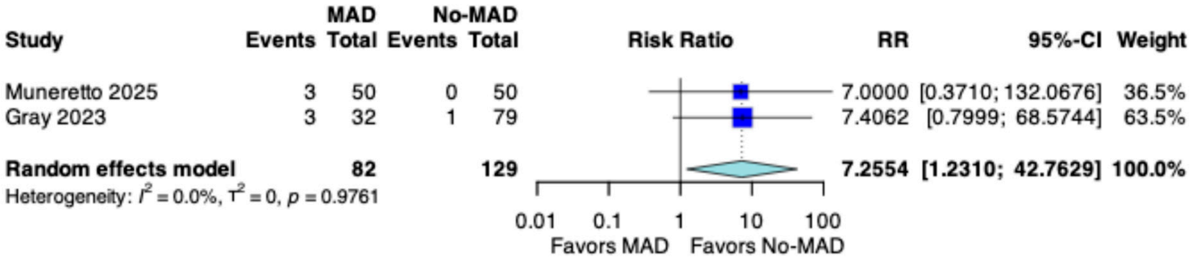

Figure S3 – Pooled estimated rate of postoperative residual mitral annular disjunction

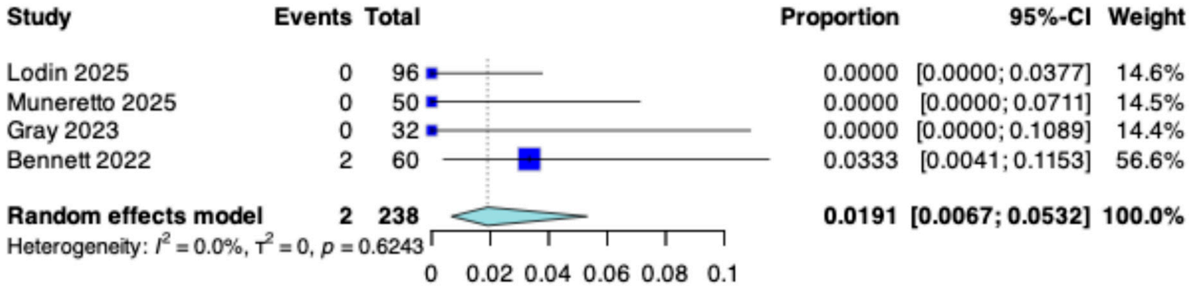

Figure S4 – Pooled estimated mean of follow-up time for MAD patients

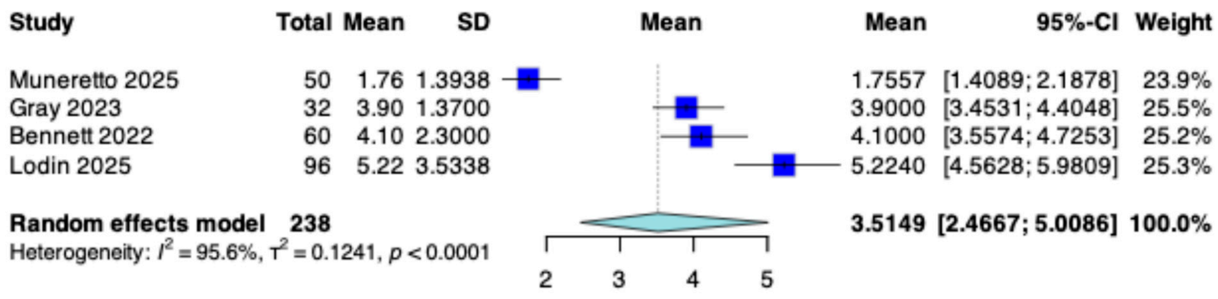

Figure S5 – Pooled estimated mean of follow-up time for No-MAD patients

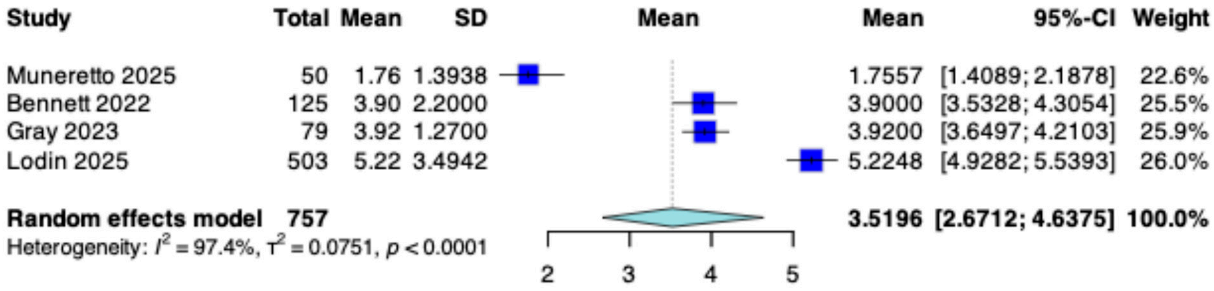

Figure S6 – Pooled estimated incidence rate ratio of follow-up ventricular arrhythmias

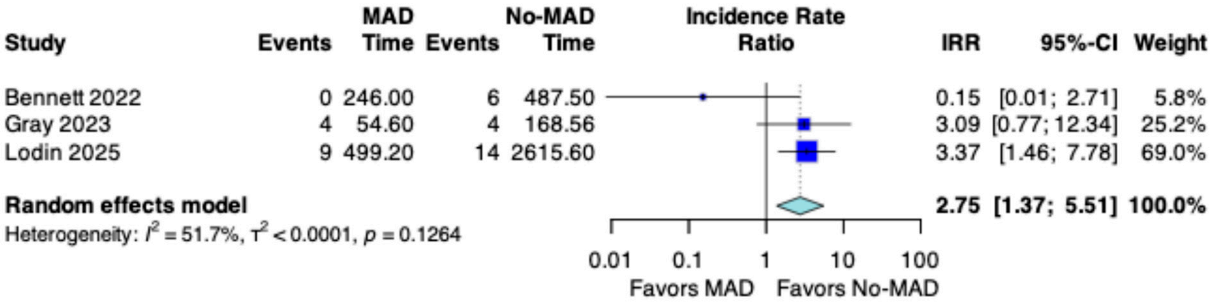

Figure S7 – Pooled estimated standardized mean difference for cardiopulmonary bypass time

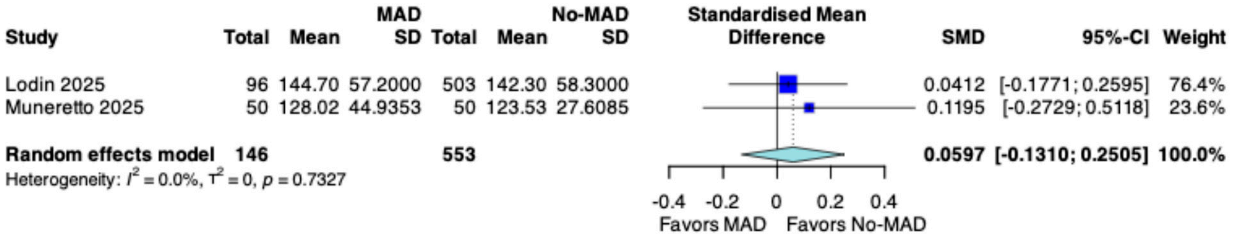

Figure S8 - Pooled estimated standardized mean difference for cross-clamp time

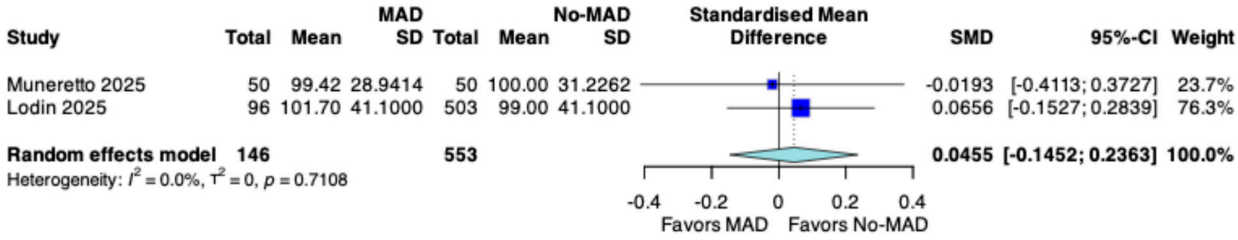

Figure S9 – Pooled estimated risk ratio of mitral valve repair

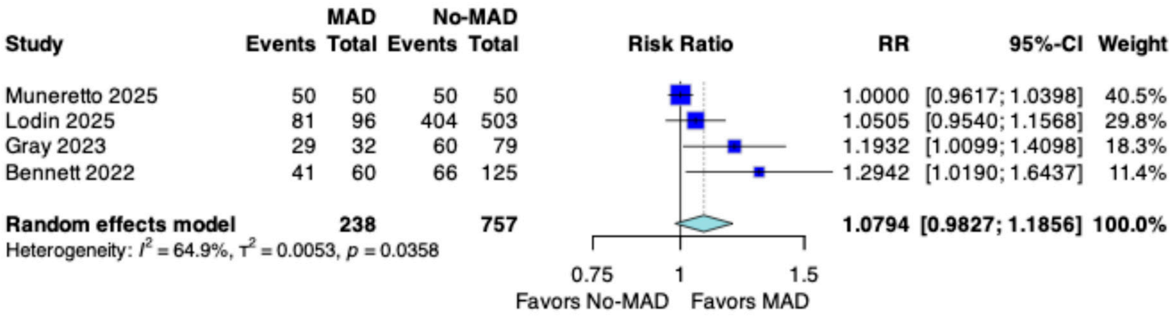

Figure S10 – Pooled estimated risk ratio of concomitant atrial fibrillation ablation

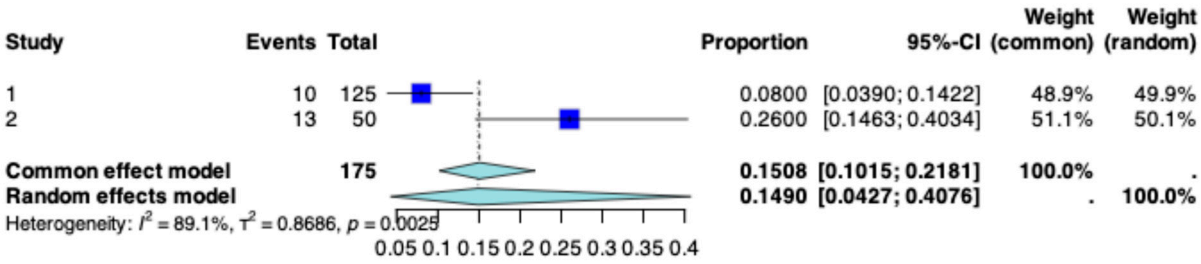

Figure S11 – Pooled estimated risk ratio of concomitant left atrial appendage occlusion

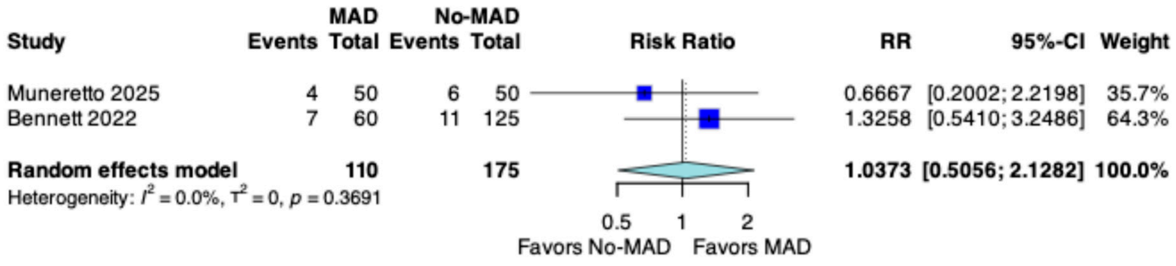

Figure S12 – Pooled estimated risk ratio of concomitant CABG

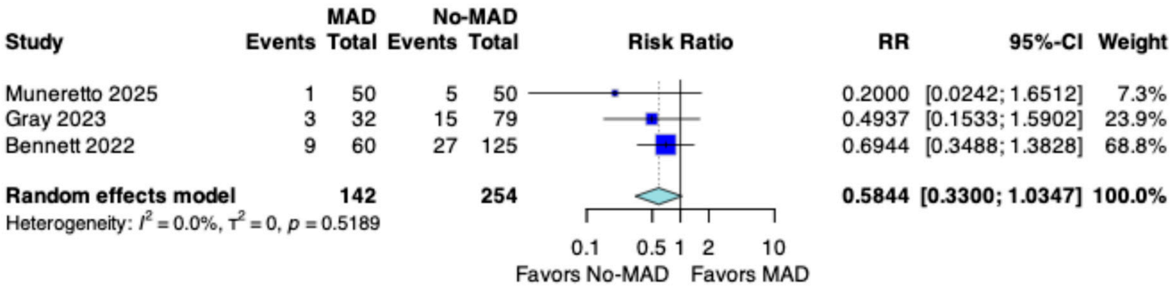

Figure S13 – Pooled estimated risk ratio of concomitant aortic surgery

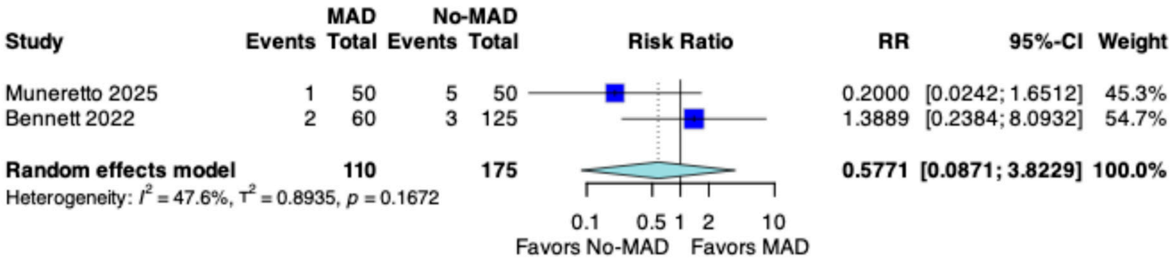

Figure S14 – Pooled estimated risk ratio of surgical revision

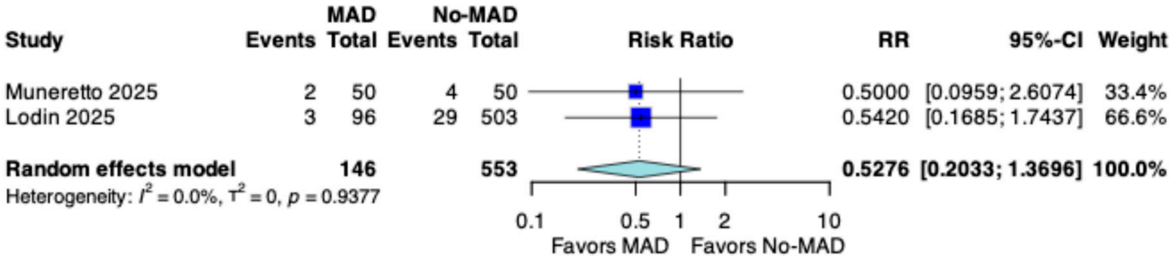

Figure S15 – Pooled estimated risk ratio of postoperative atrial fibrillation

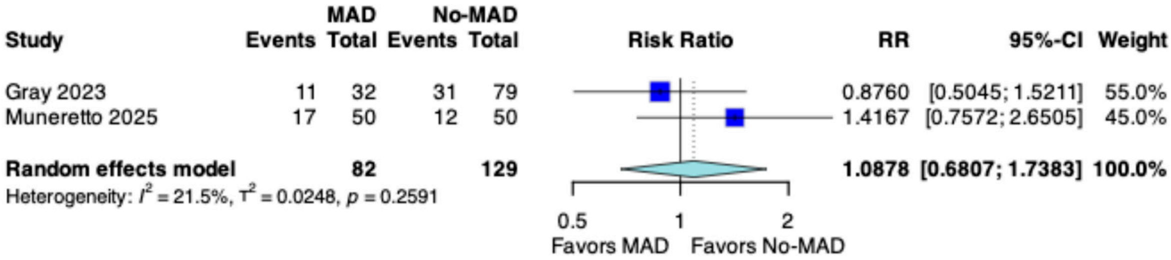

Figure S16 – Pooled estimated risk ratio of residual moderate/severe mitral regurgitation

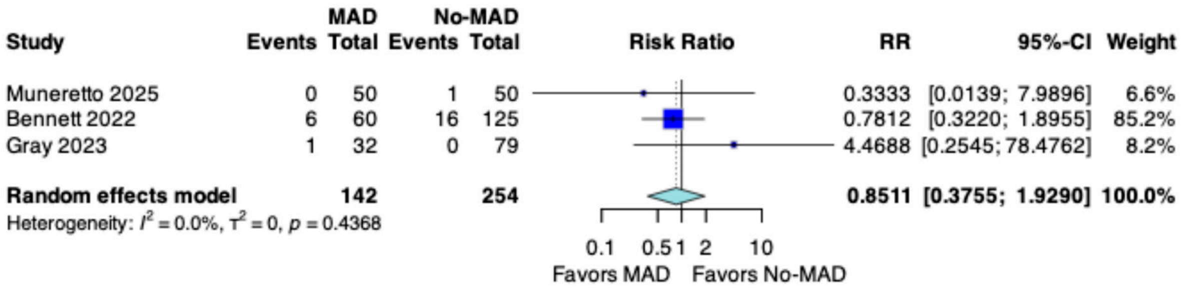

Figure S17 – Pooled estimated risk ratio of hospital mortality

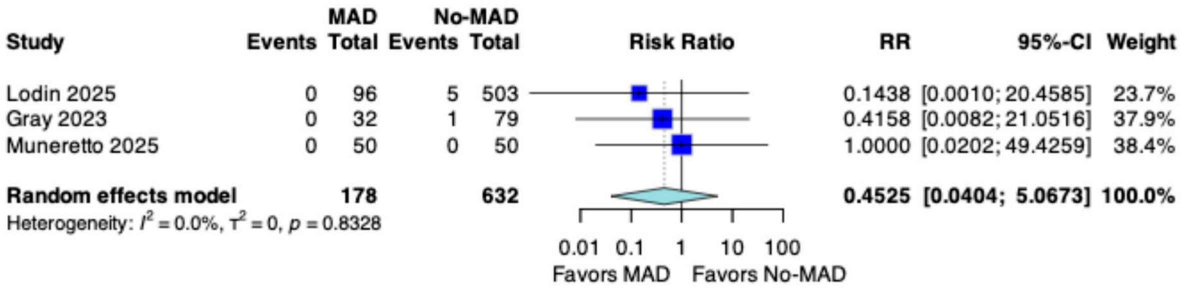

Figure S18 – Pooled estimated incidence rate ratio of follow-up mortality

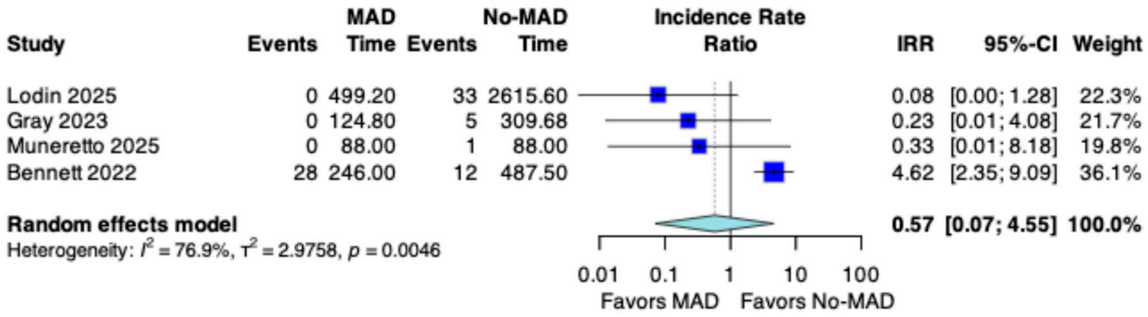

Figure S19 – Pooled estimated incidence rate ratio of cerebrovascular accidents

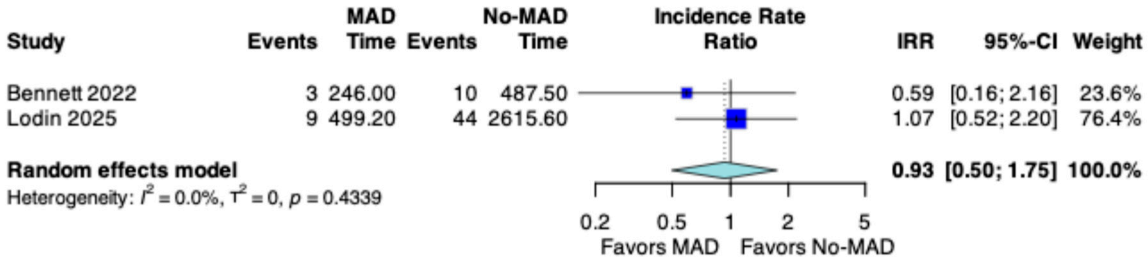

Supplement: Supplementary file 1 [file jcdd-12-00436-s001.zip › Supplementary Material.pdf]
